# A Perspective on Developing Modeling and Image Analysis Tools to Investigate Mechanosensing Proteins

**DOI:** 10.1093/icb/icad107

**Published:** 2023-08-09

**Authors:** Stephanie Ouderkirk, Alex Sedley, Mason Ong, Mary Ruth Shifflet, Quinn C Harkrider, Nathan T Wright, Callie J Miller

**Affiliations:** Department of Chemistry, James Madison University, Harrisonburg, VA 22807, USA; Department of Engineering, James Madison University, Harrisonburg, VA 22807, USA; Department of Engineering, James Madison University, Harrisonburg, VA 22807, USA; Department of Chemistry, Bridgewater College, Bridgewater, VA 22812, USA; Department of Chemistry, James Madison University, Harrisonburg, VA 22807, USA; Department of Chemistry, James Madison University, Harrisonburg, VA 22807, USA; Department of Engineering, James Madison University, Harrisonburg, VA 22807, USA

## Abstract

The shift of funding organizations to prioritize interdisciplinary work points to the need for workflow models that better accommodate interdisciplinary studies. Most scientists are trained in a specific field and are often unaware of the kind of insights that other disciplines could contribute to solving various problems. In this paper, we present a perspective on how we developed an experimental pipeline between a microscopy and image analysis/bioengineering lab. Specifically, we connected microscopy observations about a putative mechanosensing protein, obscurin, to image analysis techniques that quantify cell changes. While the individual methods used are well established (fluorescence microscopy; ImageJ WEKA and mTrack2 programs; MATLAB), there are no existing best practices for how to integrate these techniques into a cohesive, interdisciplinary narrative. Here, we describe a broadly applicable workflow of how microscopists can more easily quantify cell properties (e.g., perimeter, velocity) from microscopy videos of eukaryotic (MDCK) adherent cells. Additionally, we give examples of how these foundational measurements can create more complex, customizable cell mechanics tools and models.

## Introduction

Interdisciplinary research has the potential to tackle some of the most complex problems in society, and value in this type of research is increasing globally (Sun et al 2021). Thus, experts in different fields now have increased motivation to seek out collaborative projects. While one model for collaboration involves scientists remaining in their own discipline and handing off the data, assembly line style, to the next scientist, we favor a model where each expert teaches the other team members how they approach the problem, explains what types of experiments or analyses are reasonable, and then works creatively to connect the disciplines. In this spirit, here we walk through the rationale and the technical setup of how an engineer thinks about microscopy data.

As a secondary goal to the interdisciplinary collaboration perspective, we also present how we can use our approach to characterize a mechanosensing protein. This workflow provides experimental biologists with broadly applicable and easy-to-use computational techniques to better interrogate the mechanical properties of cells. We use already established methods and tools, but connect these methods in a way that others may find helpful in their efforts to characterize how their protein of interest correlates with cell mechanics.

Our lab is an undergraduate-only team where one group specializes in microscopy and the other group specializes in computational modeling. This particular project focuses on developing robust methods that allow students to more easily quantify aspects of cellular mechanics and motion. Until recently, such methods required significant modeling expertise. Here we assume that the reader is familiar with basic microscopy and has a basic knowledge of image analysis software, such as ImageJ. We aim to extend this knowledge through describing how and why you might want to quantify cellular properties, and how this quantitative analysis can enhance cell mechanics insights. Throughout this paper, we will reference several in-depth resources that may be of interest, but we will primarily describe basic workflow and analytical tools that we found broadly useful.

## Background

The ability of a cell to respond to stimuli is fundamental to life. External stimuli are often mechanical, which must then be converted to biochemical signaling pathways (Janmey and Miller 2011); these could include changes in proteins structure/function (Glogauer et al. 1997; del Rio et al. 2009), changes in protein localization (Janmey 1998; Miller and Davidson 2013), or gene transcription alterations (Bendig et al 2006). Regardless of the downstream, biochemical complexity, or the nuance of the biological response, the fundamental idea of how proteins initiate these changes is simple; a protein must sense a physical change and instruct an appropriate cellular reaction. The classic example of this kind of cellular mechanosensation is the cytoskeleton, in particular F-actin. When placed under high tension, F-actin bundles to form stress fibers, localizes to the area of the cellular tension, and initiates biochemical signaling cascades (Hayakawa et al. 2011; Sun et al. 2020). On the cellular level, F-actin bundles are stretched between focal adhesions, which are aligned with elongation of cells (Uttayarat et al. 2005). Thus, the function and dynamics of the F-actin is tightly coupled to cell morphology alterations. F-actin is not alone in this linkage between mechanical-induced changes in protein function and alterations to cell morphology; integrins change how they cross-dimerize when subjected to tension, which in turn modulates cell-cell adhesion and migration (Truong and Danen 2009). TRP channels are physically pulled apart under tension, resulting in a calcium influx that in turn controls cell motion (Canales et al. 2019). The physical deformation of microtubules increases the kinesin-microtubule affinity, thus slowing down the cell transport mechanism (Nasrin et al. 2021). There are believed to be many more mechanosensors yet to be discovered, and the tools we describe here help explore this type of protein/cell mechanics relationship using image analysis techniques.

Traditional mechanical studies, from engineering and physics, require quantifying descriptive parameters like Young's Modulus (which describes how a material responds elastically under tensile or compressive forces) or Poisson's ratio (which measures how a material deforms perpendicularly to an applied load); however, these values lose relevance in the context of cells or sheets of cells. The elasticity of a biological system can certainly be described and measured, but the material, by definition, is constantly changing and thus there can be no definitive Young’s Modulus for a specific cell type or for a specific condition; there is simply too much inherent variability. While the mechanical descriptive properties of a cell or tissue might provide a general approximation, biologists usually find it more useful to quantify descriptive cell properties using image analysis techniques and statistics, then correlate the results with experimental conditions. Changes in cell shape, size, confluence, and motility are the most common descriptive properties to quantify and connect to mechanical influences. Descriptions of cell shape can suggest external or internal forces distorting the cell. For example, during mitosis, cells become spherical with an associated increase in surface tension, intracellular pressure, and cortical stiffness (Taubenberger et al. 2020). Cells can also round up and decrease in size when the external milieu is soft and compliant (Rens and Merks 2020). Cell motility is quantitatively described by either the direction (Asokan et al. 2014) and/or speed that cells move (Del Alamo et al. 2007). These descriptive properties can be quantified with image analysis tools, which can then allow for the construction of more complicated models capable of predicting cell membrane tension, for example.

## Interdisciplinary collaboration example

To illustrate the usefulness of these tools, we present here our preliminary investigations of the potential mechanosensing cytoskeletal protein obscurin. Obscurin is a giant modular protein that links the cytoskeleton to the surrounding membrane system (Linke 2008; Gautel and DjinoviIc-Carugo 2016). The obscurin N-terminus consists of over 60 Ig-like domains, which as a group can flex and stretch to accommodate cellular motion (Kontrogianni-Konstantopoulous et al. 2009). The C-terminus contains a RhoGEF domain that activates RhoA and, in some isoforms, two cadherin-binding kinase domains. These signaling domains, when isolated from the rest of the protein, modulate cell adherence, and motility (Perry et al. 2014). In muscles, obscurin acts as a structural component to provide lateral organization of the sarcomere (Lange et al. 2009). In epithelial cells, obscurin knockout is linked to an epithelial to mesenchymal transition, and obscurin knockout breast epithelial cell lines experience increased motion (Stroka et al. 2016). Together this provides a picture of a protein that can both modulate cell structure and motility; however, there has been no study that examines the potential cross-talk between these structural and functional roles.

## Method 1: experimental setup and cell perimeter calculations

While software is improving in reliably detecting cells in high-quality brightfield images, it remains a challenge due to the inherently small contrast level and non-contiguous cell surfaces (Buggenthin et al. 2013; Edlund et al. 2021). Therefore, quantifying cell properties and obscurin localization currently requires the use of fluorescent probes. We had the best results using live obscurin-deficient MDCK epithelial cells treated with the Cytopainter (Abcam; Cambridge, UK) fluorescent dye, which remains well-distributed at the plasma membrane with some internalization over 10 h. The addition of Trolox was necessary to prevent free radical-induced cell death. Obscurin (∼800 kDa) is too large for efficient transfection or infection, so we infected a truncated version (160 kDa) containing the N-terminal Ig domains, a fluorescent tag, and the C-terminal region containing the RhoGEF domain, PH domain, and the ankyrin-binding region. This version localizes in the cell in a similar pattern to WT obscurin in other epithelial cells (Supplementary Material 1). Images were captured on a Nikon Eclipse Ti-2 microscope equipped with a CO_2_ chamber, heated to 37°C, and calibrated to maximize brightness and contrast between the cell membrane and the inside of the cell. Samples that were around 70% confluent provided the most reliable results. No significant photobleaching or cell toxicity was noted in our experimental setup (5–10 min between images with 5% laser power and 50 ms exposure).

To calculate cell perimeter and area, we explored multiple open-source image analysis tools. Table 1 lists a range of different programs we considered for providing quantitative descriptions of cell properties with pros/cons to consider for each. We considered ease of use and accuracy as the most important factors. Additionally, because the survival rate of our cells under the microscope greatly increased when the cells were partially confluent, we needed a tool that could accurately define the cell regions when cells were next to each other instead of surrounded by blank/black space. We chose WEKA, an ImageJ machine-learning pixel classification plugin that uses a minimal amount of manual input to segment an entire data set (Arganda-Carreras et al. 2017). In brief, the user points and clicks on small segments of the image to classify and sort these parts as either the cell membrane, cell inside, cell outside, or other regions of interest (ROIs). The user identified regions are identified by WEKA as different classes, and, at a high level, the machine-learning algorithm creates “classifiers” based on unique properties in each class, i.e., pixel brightness/intensity for our purposes. Using the “classifiers,” WEKA extrapolates and categorizes all remaining pixel regions in the image or time-lapse to these user-defined categories. In addition to its capacity to reliably segment images of cells that are near-confluent, the program also performs well with noisy images and has thorough tutorials and online resources.

**Table 1:** Collection of various Image Analysis software or techniques for quantifying cell properties.

Name of Method/Technique	Use	Software	Good for confluent cells	Post analysis adjustment	Notes	Ref
Trainable WEKA Segmentation	Boundary	ImageJ	Yes	Yes	Many video tutorials online, mostly automated	Arganda-Carreras et al. 2017
Binarizing Image	Boundary	ImageJ	No	No	Fluorescent cell membranes also show up inside cells but are hard to distinguish with a binarized image (i.e., what's inside and what's the edge/boundary)	Bovik 2009
ADAPT	Boundary	ImageJ	No	No	Little to no tutorials or resources online, cells must be isolated being a different color background	Barry 2019
Watershed	Boundary	ImageJ	No	No	Many video tutorials online, cells must be isolated on a different color background	Sage 2018
Bwboundaries	Boundary	Matlab	No	No	A few tutorials and resources online, images must be binarized before segmentation, user must be able to interpret code	Gonzalez et al. 2004
Bwtracingboundary	Boundary	Matlab	No	No	A few tutorials and resources online, images must be binarized before segmentation, must be able to interpret code	Gonzalez et al. 2004
CellOutliner	Boundary	ImageJ	No	Yes	Little to no tutorials or resources online, images are not uniform enough	Castleman 2003
Mtrack2	Velocity	ImageJ	Yes	No	Tracks cell midpoint through time, many video tutorials online, automatic	Stuurman 2003
Trackmate	Velocity	ImageJ	No	No	Cell being tracked must be a singular point, manual and automatic, many video tutorials online	Tinevez et al. 2017; Ershov et al. 2022
CellTracker	Velocity	Matlab	Yes	No	Cell being tracked must be a singular point, automatic and tracks 3D, many video tutorials online	Piccinini 2016

**Fig. 1:**
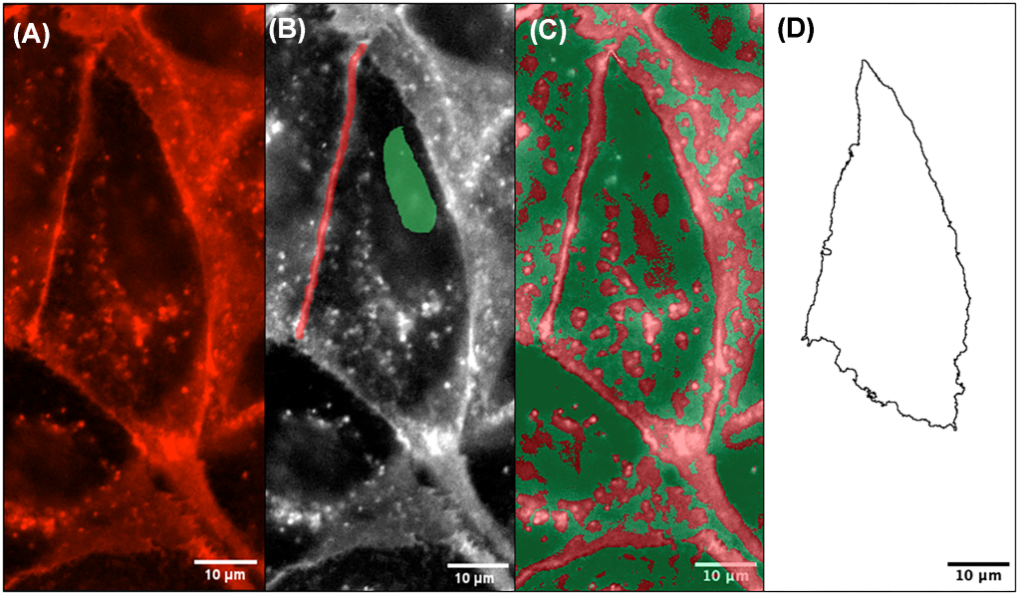
Example of input to output using WEKA for a single cell with membrane stain Cytopainter. **(A)** The initial step involves adjusting brightness and contrast from the raw microscope file to make the cell membrane areas bright but not over saturated, and the center of the cell very dim. **(B)** A grayscale image of **(A)** with user input via computer mouse pointing and clicking on the cell membrane (red) and the inside of the cell (green). **(C)** The WEKA identified areas of cell membrane and cell interior based on the user input in **(B)**. Red areas are the cell membrane, green areas are the cell interior, and this is overlaid on the grayscale image so the user can perform a visual check of accuracy. **(D)** The user can exclude incorrectly identified areas (such as the interior cell membrane red areas in **[C]**), and arrive at a final representation of the cell membrane from WEKA.

Our cells had brightly fluorescent cell membranes, dim fluorescence/punctate staining inside the cell, and nearly black regions outside the cells (Fig. 1). We identified these three example areas in WEKA via point-clicking on these regions and telling WEKA each area was a separate classifier. One common problem with this method is that the contrast between these three regions is often similar, resulting in misidentified areas. We addressed this challenge by optimizing contrast during data collection on the microscope. Postcapture optimization is only minimally effective, and requires manual adjustment of brightness and contrast. We investigated the sensitivity of WEKA to identify ROIs through increasing/decreasing brightness and contrast and compared measurements of cell areas under these conditions. For example, if we oversaturated brightness and contrast, the “bright” pixels that would be classified as cell membrane are increased, resulting a slightly larger cell area versus the “dim” version of the same image. Indeed, we found that the difference between the extremes of brightness and contrast was around 24% (Supplementary Material 2), suggesting that not only would our results be consistent across different experiments, but this percentage could be considered as standard error on any of our results.

Because many of the tools in Table 1 were built for specific purposes, potentially only distantly related to our needs, we verified WEKA's accuracy by calculating the Dice Similarity Coefficient (DSC; Dice 1945; Zou et al. 2004) between WEKA-identified cell regions and cell regions identified “by eye” or through manual methods in ImageJ (Baviskar 2011). Manually selecting each cell from a time-lapse was labor intensive so we considered only two singular microscope images with ∼20 cells for the calculation. We compared the ROIs identified by WEKA to the manual ROIs and calculated the DSC to determine the degree of overlap. A DSC of 1 would mean they were perfectly the same shape, whereas 0 would indicate no overlap. The DSC for one image with 20 ROIs was 0.84, and another image with 27 ROIs was 0.86. A good degree of overlap is considered as a DSC > 0.7 (Zijdenbos et al. 1994), so we concluded that WEKA is a reliable technique.

For scalability and statistics, one major consideration was how many cells the imaging software could count at one time. In a typical experimental image, we have anywhere from 10s to 100s of cells. A sample size analysis based on typical MDCK cell area fluctuations (20.7%; Zehnder et al. 2015) revealed that we needed ∼23 cells per experimental condition to derive meaningful statistical data. While a single-batch analysis approach is possible using WEKA, we found such a method requires significant computational memory and storage when using a typical off-the-shelf desktop computer. We therefore opted to either zoom in our timelapses to capture a few cells at a time or to take the entire field and process only 10 frames at a time and then piece together the results.

For proof-of-concept, we examined cell size using WEKA's quantification of cell area and perimeter in the presence of various drugs that affect global cell morphology (Supplementary Material 3). Blebbistatin treatment inhibits myosin II ATPase activity, resulting in decreased cell size (Tinevez et al. 2009; Liu et al. 2010; Gauthier et al. 2011; Zehnder et al. 2015). This is likely due to a disruption of cytoskeleton-induced water movement within these cells (Zehnder et al. 2015). Cytochalasin D prevents F-actin assembly and is also associated with decreased cell volume, likely through a direct disruption of the cytoskeleton (Stevenson and Begg 1994; Ting-Beall et al. 1995; Pedersen et al. 2001; Ujihara et al. 2008; Schulze et al. 2009). Y-27632 inhibits ROCK activation and also decreases cell volume, likely through changes in either cytoskeletal organization or osmotic pressure (Major et al. 2019; Di Ciano-Oliveria et al. 2003; Tinevez et al. 2009).

**Fig. 2:**
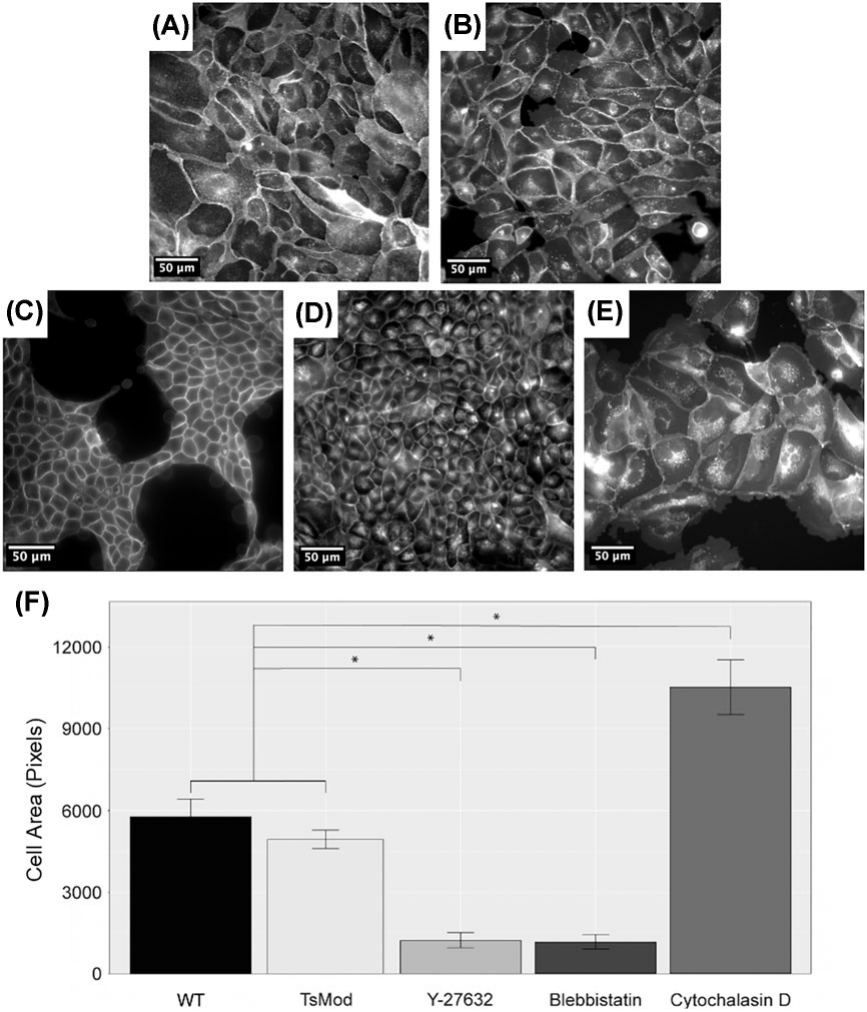
Quantification of MDCK cell size under various drug treatments using WEKA. **(A)** Example image of MDCK cells only with cell membrane stain, Cytopainter, denoted as wild type (WT). The scale bar is 50 μm for all images. **(B)** Example image of MDCK cells with Cytopainter and TsMod obscurin (not shown- in a different color channel). **(C)** Example image of MDCK cells with Y-27632 treatment. **(D)** Example image of MDCK cells with blebbistatin treatment. **(E)** Example image of MDCK cells with cytochalasinD treatment. **(F)** Bar graph comparing cell size, quantified with WEKA, between all drug treatments. (*n* = 40 for WT, Y-27632, blebbistatin, and cytochalasin D, and *n* = 32 for TsMod). Error bars are the standard error and significance (*) at *P* < 0.05.

It is difficult to see differences in cell size by eye alone (Fig. 2A–E), thus the quantification from WEKA allowed us a more sensitive and reliable measurement. We found that, in all cases, changes in cell size were significant using paired *t*-tests with *P*-values less than 0.05 (Fig. 2F). Both blebbistatin and Y-27632 decreased cell sizes as expected. CytoD treatment, however, resulted in an increase in cell size, which was unexpected. It is important to note that we are using a 2D metric of cell area to designate changes in cell size, and CytoD causes cells to decrease in volume (a 3D metric) and flatten (a 2D metric) (Schulze et al. 2009), which likely explains the increase in cell area from our results (Fig. 2F, CytoD). The addition of the short obscurin A construct does not change cell shape and perimeter significantly (Fig. 2F, TsMod vs. WT). This was expected; while obscurin interfaces with both the cytoskeleton and the surrounding membrane as a linker, but is not known to inhibit the function of either.

## Method 2: quantifying motility

**Fig. 3:**
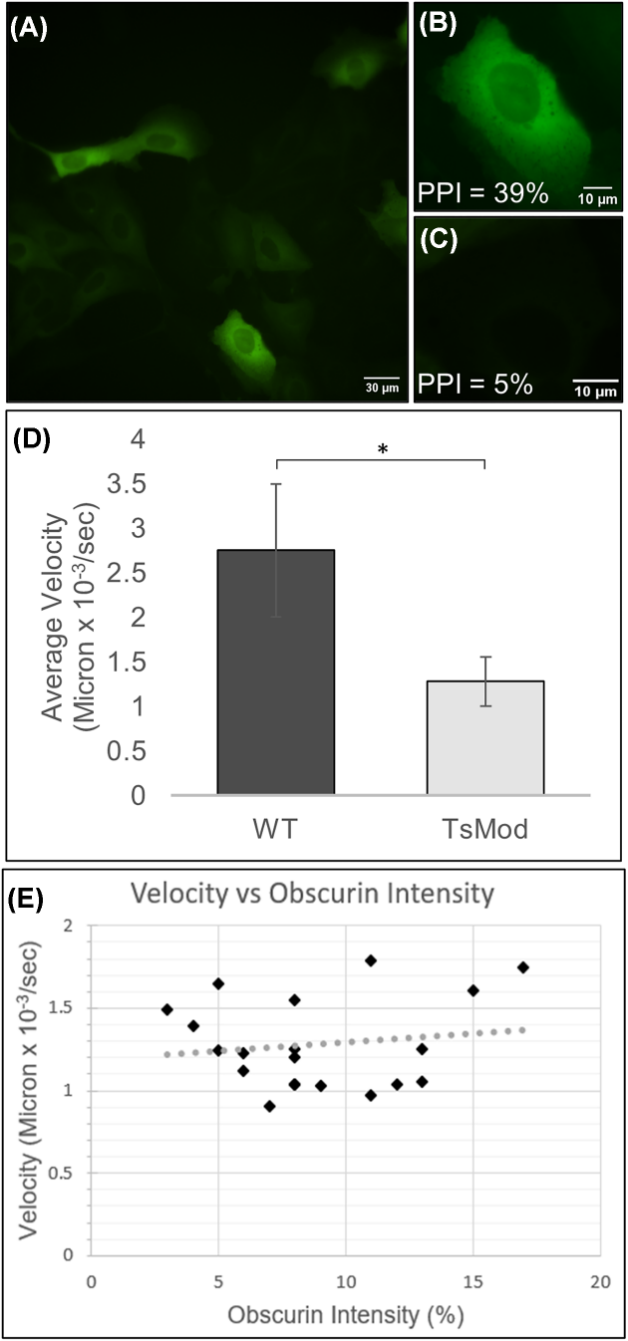
Quantifying cell velocity in MDCK cells with fluorescent obscurin. **(A)** Overexpression of fluorescent obscurin is nonuniform due to the viral delivery system. **(B)** Example of a cell that is brightly fluorescent. The % pixel intensity (PPI) is the ratio of the average pixel brightness in the individual cell over the average pixel brightness in the entire image from **A. (C)** Example of a cell that is dimly fluorescent. **(D)** Comparing the average velocity in WT MDCK cells expressing the cell membrane stain Cytopainter (*N* = 20) and the average velocity in MDCK cells with fluorescent obscurin (TsMod; *N* = 20). The change in cell velocity is statistically significant with *P* < 0.05. **(E)** Cell velocity versus obscurin intensity for 20 cells over the same experimental conditions on two different days. The R2 is 0.0228.

As with quantifying cell areas and perimeters, there are also several image analysis programs to determine cell velocities (see Table 1). We chose MTrack2, an ImageJ plugin that takes the shape of the cell identified from WEKA and uses ImageJ's “centroid” measurement tool to determine the center of the cell by averaging the number of pixels in the x direction and y direction. Once this is accomplished, MTrack2 calculates how the center point moves between frames of a time-lapse video and uses the timestamp between each frame to calculate cell velocity. Although MTrack2 has the capability to track more than one cell and find the corresponding velocity, we specifically wanted to quantify velocities in cells that were expressing fluorescent obscurin. The challenge with our infection/transfection system for fluorescent obscurin (as with many other similar systems) is that the protein is not uniformly expressed across all cells (Fig. 3A), necessitating that we crop the cell of interest for image processing. Focusing on only one cell at a time over the full time-lapse experiment (approximately 49 frames, at 10 min between frames) also allowed us to stay within the processing capabilities of our desktop computer.

We expected that obscurin overexpression in epithelial cells would decrease cell motility (Stroka et al. 2016). We thus needed to quantify the amount of obscurin in the analyzed cells. Since the overall brightness and contrast of fluorescent obscurin were not consistent between experiments, we defined the amount of obscurin present as the normalized ratio of the average pixel intensity within a single cell over the average pixel intensity of the entire image (i.e., all cells), written as a percentage of pixel intensity. We used the cell area defined by WEKA as the boundary shape for calculating the average pixel intensity of fluorescent obscurin within the boundary using the “Mean Gray Value” option in the “Measure” function in ImageJ (Fig. 3B and C). Combined with the quantified cell velocity from MTrack2, this allowed us to correlate the average cell velocity over a time lapse versus the amount of obscurin present (Fig. 3D).

Obscurin contains a RhoA-activating domain, and RhoA inhibits cell motility (Ford-Speelman et al. 2009). In addition, tumor MCF10A cells lacking obscurin exhibit increased migration (Stroka et al. 2016). In MDCK cells, which are epithelial like MCF10A but are derived from dog kidney and not human breast tissue, we found that the presence of obscurin significantly slowed down cell migration velocities (Fig. 3D). This argues that obscurin influence on motility is not cell-type specific, and in fact obscurin may generally modulate cell migration. Interesting, the amount of fluorescent obscurin present in cells was unrelated to cell velocity (Fig. 3E), arguing that it is simply the presence of obscurin, and not a dose-response, that affects motility. More research, requiring dozens of cells at each fluorescence intensity, is needed to explore these results further.

## Method 3: cell strain and obscurin

When one cell is pulling away from the plate, obscurin in the neighboring cell is often (but not always) localized near the cell that is pulling away (Supplementary Material Fig. S4), an area of presumed high membrane tension. Likewise, obscurin seems to localize to the membrane more readily in cells that are more spread out on a glass-bottomed dish, as compared to cells that are more constrained by neighboring cells. The microscopists asked whether obscurin localizing to probable areas of high cell membrane tension was a real phenomenon or whether these observations represented either confirmation bias or some other artifact.

We know that obscurin localization within cells dynamically changes, so monitoring how the cell membrane changes over time will allow us to correlate the two. We start with the simpler question: does obscurin localize to areas of high membrane strain. While a single microscopy snapshot provides almost no usable data about future or past changes in the cell membrane, a series of single time frames taken at a specified time interval can capture the dynamic movement of the cell. In engineering, how an object's length changes over time is called strain (the ratio of the change in length to the original length; Bedford and Fowler 2007); and increasing strain means the object is dilating or stretching, while decreasing strain means the object is shrinking or contracting (Craig 2011). Quantifying strain does not explain why the cell membrane is changing its length, but rather it provides a high-level estimate of the types of forces present at the cell membrane- stretch or contraction. For the cell membrane, even if an increase in length were solely the result of adding more phospholipids to the membrane, some internal mechanical force (push) is required to add this extra material to the growing membrane. If we assume that any change in an object's shape is the result of an applied force, then quantifying cell membrane strain and relating it to obscurin localization offers a pathway toward answering the larger experimental question relating obscurin localization to cellular tension.

To calculate the strain in the cell membrane, we used the quantitative data from WEKA, which indiscriminately identifies cell membranes as a series of connected (*x_i_, y_i_*) points, differentiated by index *i*. Over the course of a time lapse, cells are independently registered as ROIs in each frame, so the ROIs are “renumbered” with each image in the time lapse. For example, in frame 1 a cell might be ROI #1, but in frame 2 it's now ROI #3. This also means that WEKA uses a different number of (*x_i_, y_i_*) pairs to define ROI's in each image, so we cannot simply describe how one (*x_i_, y_i_*) node moves over time. For example, in frame 1, cell ROI #1 might have 100 nodes defining its membrane but when it becomes cell ROI #3 in frame 2, it now has 90 nodes. This necessitates the need for a reference location that will stay the same throughout the entire timelapse.

**Fig. 4:**
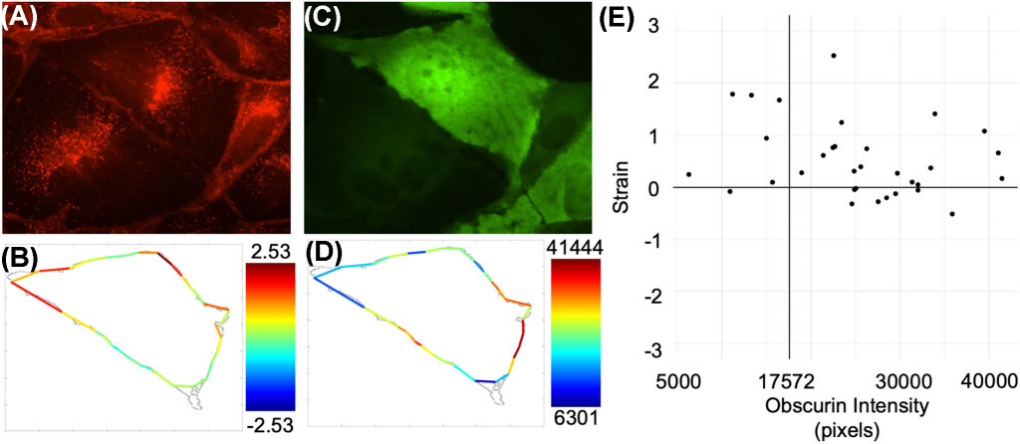
Predictive mathematical modeling can answer biological questions in microscopy, using obscurin localization and predicted membrane strain as an example. **(A)** Cell membrane stain at the end of 8 h time lapse. **(B)** Cell membrane strain from the start of the timelapse to 8 h later is calculated in MATLAB, and drawn based on the associated heatmap for low to high strain. Data for the location of the membrane comes from A. **(C)** Fluorescent obscurin expression in the same cell from A at the end of the 8 h time lapse. **(D)** Average fluorescent obscurin pixel intensity at the cell membrane in the same radial bins as B are visually represented on a heat map scale from dim (blue) to bright (red) based on the pixel value calculated in ImageJ. **(E)** A scatterplot separated into four quadrants show that membrane bins with positive strain (upper two quadrants) are more likely to also have higher obscurin pixel intensity. For determining the quadrants we determined the halfway point of the overall range of obscurin pixel values, so points to the right of the vertical black line represent the upper 50% of obscurin intenxity.

We started by determining the origin of the cell. Knowing the origin of the cell at each point in time allowed us to centralize the overall shape, to define any changes in shape as being localized to a specific part of the membrane, and to ignore any cell migration during the experiment. Using an example of a cell changing shape over the time lapse (Fig. 4A), we imported the cell membrane nodes identified by WEKA into MATLAB (see Supplementary Material files) and found the center (the average (*x, y*)). We then transformed the WEKA-identified nodes to reflect the new origin and had a MATLAB set of coordinates, (*x_i_, y_i_*) that mapped out the cell membrane, and calculated the radian angle (Equation 1):


(1) \begin{equation*}
{\theta }_i = {\tan }^{ - 1}\left( {\frac{{{y}_i}}{{{x}_i}}} \right).
\end{equation*}

We sorted our cell membrane (*x_i_, y_i_*) points into 32 bins of equal radians so that each bin spanned $2\pi /32$ radians, based on Equation 1. With the center of the cell defined and fixed, the radial bins represent portions of the cell membrane and can generally quantify changes in the cell membrane. The number of bins for discretizing the cell area can be altered as the situation warrants; more bins capture more details about small changes in the cell membrane whereas fewer bins capture larger cell membrane changes. The length of the cell membrane between points is calculated using the distance formula (Equation 2):


(2) \begin{equation*}
{d}_{i + 1} = \sqrt {{{({x}_i - {x}_{i + 1})}}^2 + {{({y}_i - {y}_{i + 1})}}^2}.
\end{equation*}

We then calculated strain as the change in length of each radial bin from the original time frame to the final time frame of the time-lapse video (Fig. 4B). Since cells dynamically change, one initial concern was that the membrane strain might be overly dependent on which frame was selected as the original, or reference, time frame. To address this, we repeated the strain calculations using different reference frames and saw no significant differences in our strain calculations. Thus, for our system, the reference for starting does not significantly affect the results.

We next quantified obscurin localization (Fig. 4C). Obscurin partially localizes to the cell membrane due to its ankyrin-binding domain and its PIP-binding PH domain, and this perimembrane area is our focus. To quantify obscurin localized to the membrane, we imported the membrane location identified from WEKA into ImageJ to quantitatively define the cell membrane, then used the “Measure” command in ImageJ to calculate the obscurin fluorescent channel pixel intensity along the cell membrane boundary. Using a custom macro for ImageJ (see supplemental code), we repeated the same radial binning from our MATLAB code to quantify the average obscurin pixel intensity in each bin (Fig. 4D). We found that dividing the perimeter into 32 bins describes obscurin's intensity along the cell perimeter reasonably well; these bins are large enough to even out much of the noise inherent in fluorescent imaging but small enough to provide sufficient subcellular detail. Results show that positive strain appears to be correlated to the upper 50% of obscurin pixel intensity datapoints (Fig. 4E), although there are not enough replicate experiments, as defined earlier, for robust statistical analysis. While this current analysis demonstrates a proof-of-concept to correlate protein localization to strain, future work will assess the correlation between the magnitude of stain and obscurin pixel intensity. This analysis can be accomplished using statistical colocalization techniques (Colocalization Analysis 2022).

Although the method presented here is at an early proof-of-concept phase, we felt it was important to include in this perspective because it demonstrates how the addition of microscopic image analysis can allow engineers to build more complex quantification methods and models. As another example of how this building process might be useful to inform future experiments, we can consider mathematical modelling of membrane tension. As a first approach, cell perimeter data from WEKA could be used to create an elastic model of cell membrane tension. In this model the membrane is modeled as a series of connected springs, where changes in membrane length over time have a corresponding change in the spring element's length, thus altering tension. This model could then be tested experimentally through microscopy techniques such as laser ablation (Kiehart et al. 2000; Fernandez-Gonzalez et al. 2009; Martin et al. 2010), AFM (Haase and Pelling 2015), micropipette aspiration (González-Bermúdez et al. 2019), drug treatments that disrupt the cytoskeletal network (Rotsch and Radmacher 2000; Miller et al. 2018), or mechanically stretching/relaxing the substrate cells are cultured on (Seriani et al. 2016). If the model is a poor predictor of experimental data, a more complex model, perhaps something like a viscoelastic model that incorporates intracellular pressure (Ishihara et al. 2013; Veldhuis et al. 2015), or cytoskeletal components like intermediate filaments (Harris et al. 2012) can be developed and tested. This iterative process allows for the eventual creation of a robust predictive model, which can then interrogate new correlations between biochemical conditions and altered cell mechanics.

## Conclusion

Here, we show a workflow of how adding cell strain data to already-established biochemical and physiological knowledge can better connect disparate data into a more unified molecular story. For our specific experimental test subject, these tools allow us to establish that overexpressing obscurin does not appear to alter cell size but does affect MDCK cell motility. Obscurin localization also appears to be correlated with membrane strain. These results are still preliminary, but point to the exciting future of testing obscurin mechanosensory capabilities directly.

Microscopists have a wealth of image data already available that could be subjected to more quantitative analyses in order to fully describe cell and protein behaviors. These data allow biologists to reach stronger conclusions about the mechanics of their given system; however, most analysis techniques are highly specialized and are likely outside the expertise of most microscopists. We have presented a path that others can use toward greater quantitative description of images. The work presented here was conducted with summer undergraduate students. Thus, these methods should be attainable for most entry-level experimentalists. With the global push toward interdisciplinary collaborations to solve complex problems, like the link between mechanosensing proteins and cell mechanics, which is complex because they are on two completely different length scales, we believe our approach demonstrates how this type of problem could be tackled with an interdisciplinary approach.

## Supplementary Material

icad107_Supplemental_File

## Data Availability

All data are either available upon reasonable request to the corresponding author, N.T.W., or located in the supplementary data.

## Notes

From the symposium “Large-scale biological phenomena arising from small-scale biophysical processes” presented at the annual meeting of the Society for Integrative and Comparative Biology virtual annual meeting, January 16–March 31, 2023
