# Supplementary material for: A Perspective on Developing Modeling and Image Analysis Tools to Investigate Mechanosensing Proteins
Source: Integr Comp Biol. 2023 Aug 9;63(6):1532–42. doi: 10.1093/icb/icad107 (PMC10755202; doi:10.1093/icb/icad107)
Supplement: icad107_Supplemental_File [file icad107_supplemental_file.docx]

**Supplement 1**


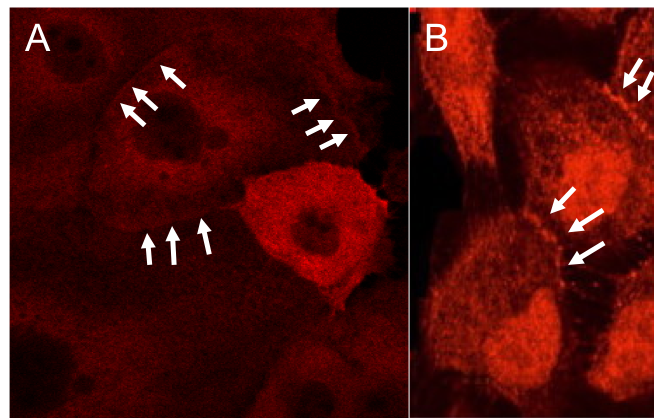


Obscurin localization in our system (A) corresponds with the expression patterns observed in the literature (B) (Figure 2C from Perry, et al, 2012). Note the punctate cellular staining and the prescence of obscurin at cell-cell contacts.

**Supplement 2**

WEKA outlines WEKA outlines

Original Image for dim image for bright image


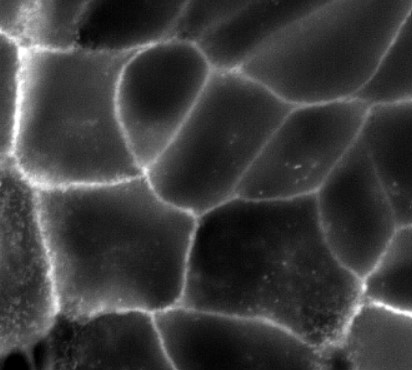

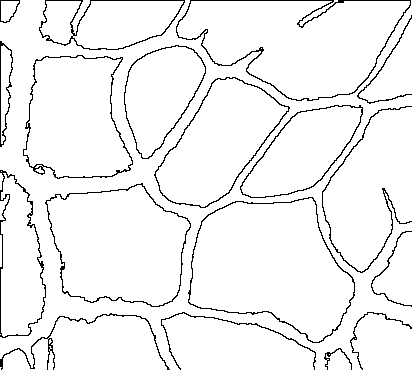

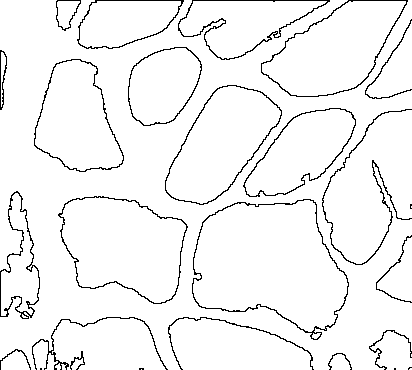


Table of WEKA calculated cell areas for a dim microscope image and the same image that is oversaturated or bright

| **Cell Identifier** | **Cell Area for dim (pixels^2^)** | **Cell Area for bright (pixels^2^)** | **Percent Difference** |
| --- | --- | --- | --- |
| 1 | 5836 | 4232 | 32% |
| 2 | 10268 | 6647 | 43% |
| 3 | 9502 | 7968 | 18% |
| 4 | 6347 | 5554 | 13% |
| 5 | 13164 | 9757 | 30% |
| 6 | 16351 | 14952 | 9% |

**Supplement 3**

Table of drug treatments and their effects on cell size and tension

| **Treatment** | **Description** | **Cell Size Effect** | **Cell Tension Effect** | **Cell Motility Effect** | **Reference** |
| --- | --- | --- | --- | --- | --- |
| Blebbistatin | Inhibits non-muscle myosin II which disrupts the cytoskeleton | Decrease | Increase | Decrease  Increase | Wang, et al, 2019; Liu, et al., 2010 |
| CytochalasinD | Disrupts f-actin network by causing depolymerization | Decrease | Decrease | Decrease | Wakatsuki, et al, 2001; Bruijns and Bult, 2001 |
| Y-27632 | Inhibits ROCK activation resulting in decrease in non-muscle myosin II contractility | Decrease | Decrease | Decrease | Tinevez, et al, 2009; Di Ciano-Oliveira, et al, 2003; Major, et al, 2019 |
| Latrunculin B | Increase actin depolymerization meaning hardly any f-actin | Decrease (rounding) | Decrease | Decrease | Pendleton and Koffer, 2001; Wakatsuki, et al, 2001 |
| Jasplakinolide | Stabilizes f-actin filaments | Reduced area, increased height | Increase (large deformations), decrease (small deformations) | Decrease / Increase (cell type dependent) | Jokhadar, et al, 2021; Ali, et al, 2021; Hayot, et al, 2006 |
| Calyculin A | Increases non-muscle myosin II contractility | Decreased (rounded) | Increase | Increase | Chartier, et al, 1991; Liu, et al, 2015 |

**Supplement 4**


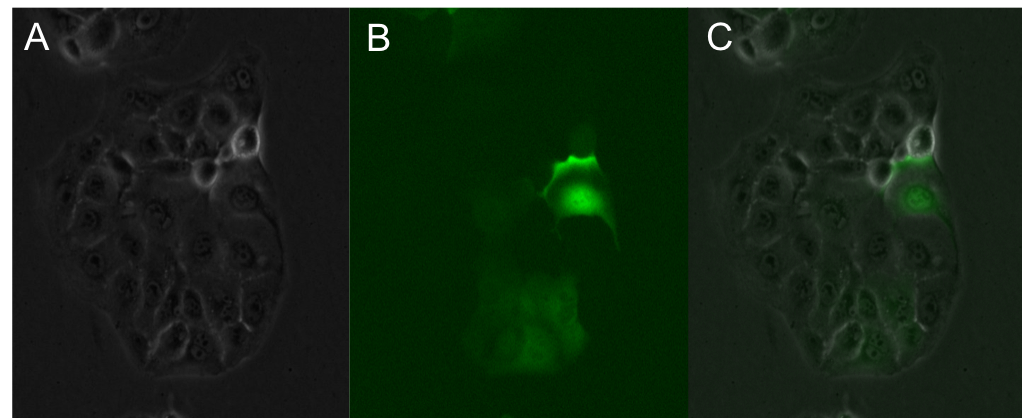


1. Phase contrast images coupled with labeled obscurin (B), collected by our undergrads. Here, the cells above the obscurin-infected cell are lifting off the dish. This produces a dramatic localization of obscurin to these membranes.

**Supplement 5**

To run our model code and capture the results seen in Figure 4, you will need XY_Coordinates_frame1.csv and XY_Coordinates_frame2.csv found in the XY_Coordinates folder, Visual_Strain.m and Visual_Obscurin.m (MATLAB codes), ObscurinFluorescenceResults.csv, XY_Coordinates_frame2_shift.csv, ObscurinPixelIntensity.txt (ImageJ macro), and the image, 4.15.22_MDCK_Marker_TsMod.nd2 - 4.15.22_MDCK_Marker_TsMod.nd2 (series 5)-SingleCell.tif. The XY_Coordinates csv files contain the (x,y) coordinate pairs from WEKA for the experimental image shown in Figure 4 at the start (Frame 1) and the end (Frame 2). The image and ImageJ macro can be used to generate a csv file of pixel brightness along the cell membrane (duplicating ObscurinFluorescenceResults.csv). As input, it’ll need XY_Coordinates_frame2_shift.csv which is the WEKA identified cell membrane shifted to match the image from the centered version of XY coordinates located in the folder.

**References**

Ali R, Mir HA, Hamid R, Shah RA, Khanday FA, Bhat SS. Jasplakinolide Attenuates Cell Migration by Impeding Alpha-1-syntrophin Protein Phosphorylation in Breast Cancer Cells. Protein J. 2021 Apr;40(2):234-244. doi: 10.1007/s10930-021-09963-y. Epub 2021 Jan 30. PMID: 33515365.

Bruijns RH, Bult H. Effects of local cytochalasin D delivery on smooth muscle cell migration and on collar-induced intimal hyperplasia in the rabbit carotid artery. Br J Pharmacol. 2001 Oct;134(3):473-83. doi: 10.1038/sj.bjp.0704281. PMID: 11588101; PMCID: PMC1572979.

Chartier L, Rankin LL, Allen RE, Kato Y, Fusetani N, Karaki H, Watabe S, Hartshorne DJ. Calyculin-A increases the level of protein phosphorylation and changes the shape of 3T3 fibroblasts. Cell Motil Cytoskeleton. 1991;18(1):26-40. doi: 10.1002/cm.970180104. PMID: 1848484.

Di Ciano-Oliveira C, Sirokmány G, Szászi K, Arthur WT, Masszi A, Peterson M, Rotstein OD, Kapus A. Hyperosmotic stress activates Rho: differential involvement in Rho kinase-dependent MLC phosphorylation and NKCC activation. Am J Physiol Cell Physiol. 2003 Sep;285(3):C555-66. doi: 10.1152/ajpcell.00086.2003. Epub 2003 May 14. PMID: 12748065.

Hayot C, Debeir O, Van Ham P, Van Damme M, Kiss R, Decaestecker C. Characterization of the activities of actin-affecting drugs on tumor cell migration. Toxicol Appl Pharmacol. 2006 Feb 15;211(1):30-40. doi: 10.1016/j.taap.2005.06.006. Epub 2005 Jul 11. PMID: 16005926.

Jokhadar, S. Z., Iturri, J, Toca-Herrera, J. L., Derganc, J. Cell stiffness under small and large deformations measured by optical tweezers and atomic force microscopy: effects of actin disrupters CK-869 and jasplakinolide. J. Phys. D: Appl. Phys. 54 124001. doi: 10.1088/1361-6463/abd0ae

Liu YJ, Le Berre M, Lautenschlaeger F, Maiuri P, Callan-Jones A, Heuzé M, Takaki T, Voituriez R, Piel M. Confinement and low adhesion induce fast amoeboid migration of slow mesenchymal cells. Cell. 2015 Feb 12;160(4):659-672. doi: 10.1016/j.cell.2015.01.007. PMID: 25679760.

Liu Z, van Grunsven LA, Van Rossen E, Schroyen B, Timmermans JP, Geerts A, Reynaert H. Blebbistatin inhibits contraction and accelerates migration in mouse hepatic stellate cells. Br J Pharmacol. 2010 Jan 1;159(2):304-15. doi: 10.1111/j.1476-5381.2009.00477.x. Epub 2009 Dec 18. PMID: 20039876; PMCID: PMC2825352.

Major LG, Holle AW, Young JL, Hepburn MS, Jeong K, Chin IL, Sanderson RW, Jeong JH, Aman ZM, Kennedy BF, Hwang Y, Han DW, Park HW, Guan KL, Spatz JP, Choi YS. Volume Adaptation Controls Stem Cell Mechanotransduction. ACS Appl Mater Interfaces. 2019 Dec 11;11(49):45520-45530. doi: 10.1021/acsami.9b19770. Epub 2019 Dec 2. PMID: 31714734.

Pendleton A, Koffer A. Effects of latrunculin reveal requirements for the actin cytoskeleton during secretion from mast cells. Cell Motil Cytoskeleton. 2001 Jan;48(1):37-51. doi: 10.1002/1097-0169(200101)48:1<37::AID-CM4>3.0.CO;2-0. PMID: 11124709.

Perry NA, Shriver M, Mameza MG, Grabias B, Balzer E, Kontrogianni-Konstantopoulos A (2012) Loss of giant obscurins promotes breast epithelial cell survival through apoptotic resistance. FASEB J. [Internet] 26:2764–2775. Available from: <http://www.fasebj.org/cgi/doi/10.1096/fj.12-205419>

Tinevez JY, Schulze U, Salbreux G, Roensch J, Joanny JF, Paluch E. Role of cortical tension in bleb growth. Proc Natl Acad Sci U S A. 2009 Nov 3;106(44):18581-6. doi: 10.1073/pnas.0903353106. Epub 2009 Oct 21. PMID: 19846787; PMCID: PMC2765453.

Wang WY, Davidson CD, Lin D, Baker BM. Actomyosin contractility-dependent matrix stretch and recoil induces rapid cell migration. Nat Commun. 2019 Mar 12;10(1):1186. doi: 10.1038/s41467-019-09121-0. PMID: 30862791; PMCID: PMC6414652.

Wakatsuki T, Schwab B, Thompson NC, Elson EL. Effects of cytochalasin D and latrunculin B on mechanical properties of cells. J Cell Sci. 2001 Mar;114(Pt 5):1025-36. doi: 10.1242/jcs.114.5.1025. PMID: 11181185.
